# Supplementary material for: Aptamers as Theragnostic Tools in Prostate Cancer
Source: Biomolecules. 2022 Jul 29;12(8):1056. doi: 10.3390/biom12081056 (PMC9406110; doi:10.3390/biom12081056)
Supplement: Supplementary file 1 [file biomolecules-12-01056-s001.zip › biomolecules-1793035-supplementary.pdf]

Supplementary Table S1. Overview of the works published in 2022 reporting the use of Aptamers in either treatment or diganostic of Cancer.

Cancer type	Year	Use	Work	DOI
glioma	2022	treatment	Engineering Macrophage Exosome Disguised Biodegradable Nanoplatform for Enhanced Sonodynamic Therapy of Glioblastoma	DOI: 10.1002/adma.202110364
breast cancer	2022	treatment	Aptamer-armed nanostructures improve the chemotherapy outcome of triple-negative breast cancer	DOI: 10.1016/j.ymthe.2022.02.004
lung cancer	2022	treatment	Targeted delivery of cancer drug paclitaxel to chordomas tumor cells via an RNA nanoparticle harboring an EGFR aptamer	DOI: 10.1016/j.colsurfb.2022.112366
Liver cancer	2022	treatment	Construction of Durvalumab/carbon nanotube/PEI/aptamer-siRNA chimera for the immunotherapy of hepatocellular carcinoma	DOI: 10.1088/1748-605X/ac5414
lung cancer	2022	treatment	Aptamer-functionalized targeted siRNA delivery system for tumor immunotherapy	DOI: 10.1088/1748-605X/ac5415
different types of cancer	2022	diagnostic	Multiplexed miRNA detection based on target-triggered transcription of multicolor fluorogenic RNA aptamers	DOI: 10.1016/j.bios.2022.114071
different types of cancer	2022	diagnostic	Unbiased Enrichment of Circulating Tumor Cells Via DNAzyme-Catalyzed Proximal Protein Biotinylation	DOI: 10.1021/acs.nanolett.1c04583
leukemia	2022	treatment	A targeting delivery system for effective genome editing in leukemia cells to reverse malignancy	DOI: 10.1016/j.jconrel.2022.02.012
prostate cancer	2022	diagnostic	Detection of prostate specific antigen in whole blood by microfluidic chip integrated with dielectrophoretic separation and electrochemical sensing	DOI: 10.1016/j.bios.2022.114057
esophagus cancer	2022	treatment	Nucleic acid aptamer controls mycoplasma infection for inhibiting the malignancy of esophageal squamous cell carcinoma	DOI: 10.1016/j.ymthe.2022.02.018
ovarian cancer	2022	diagnostic	Engineering a G-quadruplex-based logic gate platform for sensitive assay of dual biomarkers of ovarian cancer	DOI: 10.1016/j.aca.2022.339559
lung cancer	2022	treatment	A novel DNA aptamer targeting lung cancer stem cells exerts a therapeutic effect by binding and neutralizing Annexin A2	DOI: 10.1016/j.omtn.2022.01.012
different types of cancer	2022	diagnostic	Simultaneous Imaging of Dual microRNAs in Cancer Cells through Catalytic Hairpin Assembly on a DNA Tetrahedron	DOI: 10.1021/acsami.1c23227
gastric cancer	2022	diagnostic	A novel signal amplification tag to develop rapid and sensitive aptamer-based biosensors	DOI: 10.1016/j.bioelechem.2022.108087
breast cancer	2022	diagnostic	A high-performance electrochemical aptasensor based on graphene-decorated rhodium nanoparticles to detect HER2-ECD oncomarker in liquid biopsy	DOI: 10.1038/s41598-022-07230-3
pancreatic cancer	2022	treatment	Anticancer effect of locally applicable aptamer-conjugated gemcitabine-loaded atelocollagen patch in pancreatic cancer patient-derived xenograft models	DOI: 10.1111/cas.15318
melanoma	2022	treatment	Dacarbazine-Loaded Targeted Polymeric Nanoparticles for Enhancing Malignant Melanoma Therapy	DOI: 10.3389/fbioe.2022.847901
colon cancer	2022	treatment	Selection of CD133-targeted DNA aptamers for the efficient and specific therapy of colorectal cancer	DOI: 10.1039/d1tb02729h

different types of cancer	2022	treatment	Novel Complex of PD-L1 Aptamer and Albumin Enhances Antitumor Efficacy In Vivo	DOI: 10.3390/molecules27051482
colon cancer	2022	diagnostic	In Vivo Evaluation of Sgc8-c Aptamer as a Molecular Imaging Probe for Colon Cancer in a Mouse Xenograft Model	DOI: 10.3390/ijms23052466
breast cancer	2022	treatment	Dual-Targeted Self-Assembled DNA Hydrogels Decorated With Multivalent Aptamers Loaded With DOX for Anticancer Therapy	DOI: 10.3389/fphar.2022.807498
breast cancer	2022	diagnostic	Site-specific DNA functionalization through the tetrazene-forming reaction in ionic liquids	DOI: 10.1039/d1sc05204g
different types of cancer	2022	diagnostic	ATP-Triggered Intracellular In Situ Aggregation of a Gold-Nanoparticle-Equipped Triple-Helix Molecular Switch for Fluorescence Imaging and Photothermal Tumor Therapy	DOI: 10.1021/acs.langmuir.1c03331
different types of cancer	2022	diagnostic	A high-integrated DNA biocomputing platform for MicroRNA sensing in living cells	DOI: 10.1016/j.bios.2022.114183
lung cancer	2022	treatment	Targeting lung cancer cells with MUC1 aptamer-functionalized PLA-PEG nanocarriers	DOI: 10.1038/s41598-022-08759-z
different types of cancer	2022	diagnostic	Surface plasmon resonance aptasensor for soluble ICAM-1 protein in blood samples	DOI: 10.1039/d1an02332b
breast cancer	2022	diagnostic	Aptamer Targets Triple-Negative Breast Cancer through Specific Binding to Surface CD49c	DOI: 10.3390/cancers14061570
breast cancer	2022	treatment	Selective Photo-Assisted Eradication of Triple-Negative Breast Cancer Cells through Aptamer Decoration of Doped Conjugated Polymer Nanoparticles	DOI: 10.3390/pharmaceutics14030626
different types of cancer	2022	treatment	Structure-switchable aptamer-arranged reconfigurable DNA nanonetworks for targeted cancer therapy	DOI: 10.1016/j.nano.2022.102553
different types of cancer	2022	diagnostic	Multibranched Linear DNA-Controlled Assembly of Silver Nanoclusters and Their Applications in Aptamer-Based Cell Recognition	DOI: 10.1021/acsami.1c24547
Liver cancer	2022	treatment	Inhibitory effect of aptamer-carbon dot nanomaterial-siRNA complex on the metastasis of hepatocellular carcinoma cells by interfering with FMRP	DOI: 10.1016/j.ejpb.2022.03.013
different types of cancer	2022	diagnostic	Aptamer-Initiated Catalytic Hairpin Assembly Fluorescence Assay for Universal, Sensitive Exosome Detection	DOI: 10.1021/acs.analchem.2c00231
different types of cancer	2022	treatment	Activatable Dual Cancer-Related RNA Imaging and Combined Gene-Chemotherapy through the Target-Induced Intracellular Disassembly of Functionalized DNA Tetrahedron	DOI: 10.1021/acs.analchem.2c00364
different types of cancer	2022	treatment	Binding affinity and conformation of a conjugated AS1411 aptamer at a cationic lipid bilayer interface	DOI: 10.1039/d1cp05753g
different types of cancer	2022	treatment	Disruption of dual homeostasis by a metal-organic framework nanoreactor for ferroptosis-based immunotherapy of tumor	DOI: 10.1016/j.biomaterials.2022.121502

different types of cancer	2022	treatment	Triple-color fluorescence co-localization of PD-L1-overexpressing cancer exosomes	DOI: 10.1007/s00604-022-05278-6
esophagus cancer	2022	diagnostic	Identification of DNA aptamers that specifically targets EBV + nasopharyngeal carcinoma via binding with EphA2/CD98hc complex	DOI: 10.1016/j.bbrc.2022.03.157
different types of cancer	2022	treatment	Design and synthesis of aptamer-cyclometalated iridium(III) complex conjugate targeting cancer cells	DOI: 10.1016/j.ejmech.2022.114335
different types of cancer	2022	treatment	Optimization of Short RNA Aptamers for TNBC Cell Targeting	DOI: 10.3390/ijms23073511
bladder cancer	2022	diagnostic	Elucidation of CKAP4-remodeled cell mechanics in driving metastasis of bladder cancer through aptamer-based target discovery	DOI: 10.1073/pnas.2110500119
breast cancer	2022	diagnostic	Dual rolling circle amplification-enabled ultrasensitive multiplex detection of exosome biomarkers using electrochemical aptasensors	DOI: 10.1016/j.aca.2022.339762
leukemia	2022	diagnostic	Rolling circle amplification assisted dual signal amplification colorimetric biosensor for ultrasensitive detection of leukemia-derived exosomes	DOI: 10.1016/j.talanta.2022.123444
breast cancer	2022	treatment	Cell-directed aptamer therapeutic targeting for cancers including those within the central nervous system	DOI: 10.1080/2162402X.2022.2062827
different types of cancer	2022	diagnostic and treatment	Novel DNA Aptamer for CYP24A1 Inhibition with Enhanced Antiproliferative Activity in Cancer Cells	DOI: 10.1021/acsami.1c22965
different types of cancer	2022	treatment	Dual-aptamer-engineered M1 macrophage with enhanced specific targeting and checkpoint blocking for solid-tumor immunotherapy	DOI: 10.1016/j.ymthe.2022.04.015
prostate cancer	2022	treatment	High eEF1A1 Protein Levels Mark Aggressive Prostate Cancers and the In Vitro Targeting of eEF1A1 Reveals the eEF1A1-actin Complex as a New Potential Target for Therapy	DOI: 10.3390/ijms23084143
Liver cancer	2022	diagnostic	Structure based innovative approach to analyze aptaprobe-GPC3 complexes in hepatocellular carcinoma	DOI: 10.1186/s12951-022-01391-z
breast cancer	2022	treatment	Enhanced Functional Properties of Three DNA Origami Nanostructures as Doxorubicin Carriers to Breast Cancer Cells	DOI: 10.1021/acsabm.2c00114
breast cancer	2022	treatment	Electrochemical detection of human epidermal growth factor receptor 2 using an aptamer on cobalt phthalocyanines - Cerium oxide nanoparticle conjugate	DOI: 10.1016/j.bioelechem.2022.108146
different types of cancer	2022	treatment	Rationally Screened and Designed ABCG2-Binding Aptamers for Targeting Cancer Stem Cells and Reversing Multidrug Resistance	DOI: 10.1021/acs.analchem.2c00863
different types of cancer	2022	diagnostic and treatment	Acidic microenvironment triggered in situ assembly of activatable three-arm aptamer nanoclaw for contrast-enhanced imaging and tumor growth inhibition in vivo	DOI: 10.7150/thno.72028
different types of cancer	2022	treatment	Inhibition of Human Urokinase-Type Plasminogen Activator (uPA) Enzyme Activity and Receptor Binding by DNA Aptamers as Potential Therapeutics through Binding to the Different Forms of uPA	DOI: 10.3390/ijms23094890

glioma	2022	treatment	A Combined Effect of G-Quadruplex and Neuro-Inducers as an Alternative Approach to Human Glioblastoma Therapy	DOI: 10.3389/fonc.2022.880740
breast cancer	2022	treatment	Multifunctional Theranostic Nanoparticles for Enhanced Tumor Targeted Imaging and Synergistic FUS/Chemotherapy on Murine 4T1 Breast Cancer Cell	DOI: 10.2147/IJN.S360161
different types of cancer	2022	diagnostic	Carbon Material Hybrid Construction on an Aptasensor for Monitoring Surgical Tumors	DOI: 10.1155/2022/9740784
breast cancer	2022	diagnostic and treatment	Synthesis of a targeted, dual pH and redox-responsive nanoscale coordination polymer theranostic against metastatic breast cancer in vitro and in vivo	DOI: 10.1080/17425247.2022.2083602
different types of cancer	2022	treatment	In silico SELEX screening and statistical analysis of newly designed 5mer peptide-aptamers as Bcl-xl inhibitors using the Taguchi method	DOI: 10.1016/j.compbiomed.2022.105632
different types of cancer	2022	diagnostic	Chaperone Copolymer Assisted G-Quadruplex-Based Signal Amplification Assay for Highly Sensitive Detection of VEGF	DOI: 10.3390/bios12050262
lung cancer	2022	diagnostic	Rapid Capturing and Chemiluminescent Sensing of Programmed Death Ligand-1 Expressing Extracellular Vesicles	DOI: 10.3390/bios12050281
different types of cancer	2022	treatment	CD133-Functionalized Gold Nanoparticles as a Carrier Platform for Telaglenastat (CB-839) against Tumor Stem Cells	DOI: 10.3390/ijms23105479
different types of cancer	2022	treatment	Aptamer-Gemcitabine Conjugates with Enzymatically Cleavable Linker for Targeted Delivery and Intracellular Drug Release in Cancer Cells	DOI: 10.3390/ph15050558
different types of cancer	2022	treatment	High Performance Gold Nanorods@DNA Self-Assembled Drug-Loading System for Cancer Thermo-Chemotherapy in the Second Near-Infrared Optical Window	DOI: 10.3390/pharmaceutics14051110
different types of cancer	2022	treatment	Gold nanorods/tetrahedral DNA composites for chemo-photothermal therapy	DOI: 10.1093/rb/rbac032
lung cancer	2022	treatment	Efficacy of newly discovered DNA aptamers targeting AXL in a lung cancer cell with acquired resistance to Erlotinib	DOI: 10.21037/tcr-20-2447
different types of cancer	2022	diagnostic and treatment	Accurate Isolation of Circulating Tumor Cells via a Heterovalent DNA Framework Recognition Element-Functionalized Microfluidic Chip	DOI: 10.1021/acssensors.1c02692
different types of cancer	2022	treatment	Cell-Selective Encapsulation within Metal-Organic Framework Shells via Precursor-Functionalized Aptamer Identification for Whole-Cell Cancer Vaccine	DOI: 10.1002/smtd.202101391
gastric cancer	2022	diagnostic	Dual-Aptamer-Targeted Immunomagnetic Nanoparticles to Accurately Explore the Correlations between Circulating Tumor Cells and Gastric Cancer.	DOI: 10.1021/acsami.1c22720
different types of cancer	2022	diagnostic	Membrane Protein and Extracellular Acid Heterogeneity-Driven Amplified DNA Logic Gate Enables Accurate and Sensitive Identification of Cancer Cells	DOI: 10.1021/acs.analchem.1c04347

different types of cancer	2022	treatment	Aptamers Entirely Built from Therapeutic Nucleoside Analogues for Targeted Cancer Therapy	DOI: 10.1021/jacs.1c09574
breast cancer	2022	treatment	Biomimetic Nanoerythrosome-Coated Aptamer-DNA Tetrahedron/Maytansine Conjugates: pH-Responsive and Targeted Cytotoxicity for HER2-positive Breast Cancer	DOI: 10.1002/adma.202109609
bladder cancer	2022	treatment	Development of novel aptamer-based targeted chemotherapy for bladder cancer	DOI: 10.1158/0008-5472.CAN-21-2691
different types of cancer	2022	diagnostic	Autofluorescence free detection of carcinoembryonic antigen in pleural effusion by persistent luminescence nanoparticle-based aptasensors	DOI: 10.1016/j.aca.2021.339408
different types of cancer	2022	treatment	Synthesis and characterization of novel ssDNA X-aptamers targeting Growth Hormone Releasing Hormone (GHRH).	DOI: 10.1371/journal.pone.0260144
different types of cancer	2022	diagnostic	Translating cancer exosomes detection into the color change of phenol red based on target-responsive DNA microcapsules	DOI: 10.1016/j.aca.2021.339357
different types of cancer	2022	diagnostic	In-Situ Integration of 3D C-MEMS Microelectrodes with Bipolar Exfoliated Graphene for Label-Free Electrochemical Cancer Biomarkers Aptasensor	DOI: 10.3390/mi13010104
lymphoma	2022	treatment	T908 Polymeric Micelles Improved the Uptake of Sgc8-c Aptamer Probe in Tumor-Bearing Mice: A Co-Association Study between the Probe and Preformed Nanostructures	DOI: 10.3390/ph15010015
leukemia	2022	treatment	Lipid Specific Membrane Interaction of Aptamers and Cytotoxicity	DOI: 10.3390/membranes12010037
breast cancer	2022	treatment	Complement-Mediated Selective Tumor Cell Lysis Enabled by Bi-Functional RNA Aptamers	DOI: 10.3390/genes13010086
prostate cancer	2022	diagnostic	Crystal-reconstructed BiVO 4 semiconductor photoelectrochemical sensor for ultra-sensitive tumor biomarker detection	DOI: 10.1039/d1tb02576g
different types of cancer	2022	diagnostic	Peptide-Conjugated Silver Nanoparticles for the Colorimetric Detection of the Oncoprotein Mdm2 in Human Serum	DOI: 10.1002/cplu.202200043
breast cancer	2022	diagnostic	An Ultrasensitive Strand Displacement Signal Amplification-Assisted Synchronous Fluorescence Assay for Surface Proteins of Small Extracellular Vesicle Analysis and Cancer Identification	DOI: 10.1021/acs.analchem.1c04122
different types of cancer	2022	treatment	Mesoporous silica@chitosan@gold nanoparticles as "on/off" optical biosensor and pH-sensitive theranostic platform against cancer	DOI: 10.1016/j.ijbiomac.2022.01.063
different types of cancer	2022	diagnostic	High sensitivity detection of tumor cells in biological samples using a multivalent aptamer strand displacement strategy	DOI: 10.1039/d1an01949j
different types of cancer	2022	diagnostic	A dual-modal aptasensor based on a multifunctional acridone derivate for exosomes detection	DOI: 10.1016/j.aca.2021.339279
Liver cancer	2022	diagnostic	A highly sensitive electrochemical cytosensor based on a triple signal amplification strategy using both nanozyme and DNAzyme	DOI: 10.1039/d1tb02545g

different types of cancer	2022	diagnostic	A dual-recognition-controlled electrochemical biosensor for accurate and sensitive detection of specific circulating tumor cells	DOI: 10.1016/j.bios.2022.113973
different types of cancer	2022	diagnostic	An aptamer-tethered DNA origami amplifier for sensitive and accurate imaging of intracellular microRNA	DOI: 10.1039/d1nr06399e
breast cancer	2022	treatment	RNA Hydrogel Combined with MnO 2 Nanoparticles as a Nano-Vaccine to Treat Triple Negative Breast Cancer	DOI: 10.3389/fchem.2021.797094
prostate cancer	2022	diagnostic	Synergetic PtNP@Co 3 O 4 hollow nanopolyhedrals as peroxidase-like nanozymes for the dual-channel homogeneous biosensing of prostate-specific antigen	DOI: 10.1007/s00216-021-03827-1
different types of cancer	2022	diagnostic	DNA-Engineered iron-based metal-organic framework bio-interface for rapid visual determination of exosomes	DOI: 10.1016/j.jcis.2021.12.133
different types of cancer	2022	diagnostic	A competitive assay for the detection of a 16-mer peptide from α1 chain of human collagen XI	DOI: 10.1016/j.talanta.2021.123196
colon cancer	2022	treatment	Smart delivery of epirubicin to cancer cells using aptamer-modified ferritin nanoparticles	DOI: 10.1080/1061186X.2022.2025600
colon cancer	2022	diagnostic	Colorimetric immunosensor constructed using 2D metal-organic framework nanosheets as enzyme mimics for the detection of protein biomarkers	DOI: 10.1039/d1tb02192c
breast cancer	2022	diagnostic	Ultrasensitive DNA-Biomacromolecule Sensor for the Detection Application of Clinical Cancer Samples	DOI: 10.1002/advs.202102804
Liver cancer	2022	treatment	Development of CD44E/s dual-targeting DNA aptamer as nanoprobe to deliver treatment in hepatocellular carcinoma	DOI: 10.7150/ntno.62639
breast cancer	2022	diagnostic	Classification of breast cancer by a gold nanoparticle based multicolor fluorescent aptasensor	DOI: 10.1016/j.jcis.2021.12.039
cervical cancer	2022	treatment	Targeting drug delivery and efficient lysosomal escape for chemo-photodynamic cancer therapy by a peptide/DNA nanocomplex	DOI: 10.1039/d1tb02441h
glioma	2022	treatment	Membrane-Decorated Exosomes for Combination Drug Delivery and Improved Glioma Therapy	DOI: 10.1021/acs.langmuir.1c02500
melanoma	2022	treatment	CD71-Specific Aptamer Conjugated with Monomethyl Auristatin E for the Treatment of Uveal Melanoma	DOI: 10.1021/acsami.1c13980
different types of cancer	2022	diagnostic	MOF-derivated MnO@C nanocomposite with bidirectional electrocatalytic ability as signal amplification for dual-signal electrochemical sensing of cancer biomarker	DOI: 10.1016/j.talanta.2021.123150
different types of cancer	2022	diagnostic	A pretreatment-free electrical capacitance biosensor for exosome detection in undiluted serum	DOI: 10.1016/j.bios.2021.113872
breast cancer	2022	diagnostic	MUC1 detection and in situ imaging method based on aptamer conformational switch and hybridization chain reaction	DOI: 10.1016/j.talanta.2021.123129
breast cancer	2022	treatment	Carboxylated chitosan-mediated improved efficacy of mesoporous silica nanoparticle-based targeted drug delivery system for breast cancer therapy	DOI: 10.1016/j.carbpol.2021.118822

breast cancer	2022	treatment	Design and synthesis of a star-like polymeric micelle modified with AS1411 aptamer for targeted delivery of camptothecin for cancer therapy	DOI: 10.1016/j.ijpharm.2021.121346
different types of cancer	2022	treatment	A pH-Responsive Nanoplatform Based on Fluorescent Conjugated Polymer Dots for Imaging-Guided Multitherapeutics Delivery and Combination Cancer Therapy	DOI: 10.1021/acsbiomaterials.1c01244
breast cancer	2022	treatment	Smart drug delivery of p-Coumaric acid loaded aptamer conjugated starch nanoparticles for effective triple-negative breast cancer therapy	DOI: 10.1016/j.ijbiomac.2021.11.170
Liver cancer	2022	treatment	Aptamer-mediated doxorubicin delivery reduces HCC burden in 3D organoids model	DOI: 10.1016/j.jconrel.2021.11.036
different types of cancer	2022	diagnostic	An abiotic fluorescent probe for the detection and quantification of carcinoembryonic antigen	DOI: 10.1016/j.bioorg.2021.105490
prostate cancer	2022	diagnostic	Simultaneous detection of dual biomarkers using hierarchical MoS 2 nanostructuring and nano-signal amplification-based electrochemical aptasensor toward accurate diagnostic of prostate cancer	DOI: 10.1016/j.bios.2021.113797
different types of cancer	2022	diagnostic	Aptamers targeting a tumor-associated extracellular matrix component: The human mature collagen XIα1	DOI: 10.1016/j.aca.2021.339206
breast cancer	2022	treatment	Targeted immunotherapy of triple-negative breast cancer by aptamer-engineered NK cells	DOI: 10.1016/j.biomaterials.2021.121259
breast cancer	2022	diagnostic	Luminous MoS 2 nanosheet-based electrochemiluminescence biosensor with biomimetic vesicle for miRNA-210 detection	DOI: 10.1016/j.talanta.2021.122969
different types of cancer	2022	diagnostic	An electrochemical aptasensor for highly sensitive detection of CEA based on exonuclease III and hybrid chain reaction dual signal amplification	DOI: 10.1016/j.bioelechem.2021.107986
different types of cancer	2022	diagnostic	Research of exosome in bone metastasis through dual aptamer recognition based entropy-driven amplification	DOI: 10.1016/j.ab.2021.114433
breast cancer	2022	diagnostic	Gold nanoparticles conjugated with DNA aptamer for photoacoustic detection of human matrix metalloproteinase-9	DOI: 10.1016/j.pacs.2021.100307
different types of cancer	2022	treatment	VEGF aptamer/i-motif-grafted multi-functional SPION nanocarrier for chemotherapeutic/phototherapeutic synergistic research	DOI: 10.1177/08853282211049620
colon cancer	2022	treatment	A new method based on guanine rich aptamer structural change for carcinoembryonic antigen detection	DOI: 10.1016/j.talanta.2021.122867
different types of cancer	2022	treatment	pH-Responsive DNA nanoassembly for detection and combined therapy of tumor	DOI: 10.1016/j.bios.2021.113654
breast cancer	2022	treatment	MXene-based cytosensor for the detection of HER2-positive cancer cells using CoFe 2 O 4@Ag magnetic nanohybrids conjugated to the HB5 aptamer	DOI: 10.1016/j.bios.2021.113626
osteosarcoma	2022	diagnostic	In vitro selection of DNA aptamers against human osteosarcoma	DOI: 10.1007/s10637-021-01161-y

different types of cancer	2022	treatment	Designing of RNA aptamer against DNA binding domain of the glucocorticoid receptor: A response element-based in-silico approach	DOI: 10.1080/07391102.2020.1822918
prostate cancer	2022	treatment	Pheno-SELEX: Engineering Anti-Metastatic Aptamers through Targeting the Invasive Phenotype Using Systemic Evolution of Ligands by Exponential Enrichment	DOI: 10.3390/bioengineering8120212
